# Oxytocin modulates insulin and GLP-1 secretion in pancreatic islets

**DOI:** 10.18632/aging.206244

**Published:** 2025-05-01

**Authors:** Kasumi Hattori, Masaru Shimizu, Megumi Yamachi, Kiichi Tezuka, Toru Fukushima, Syoko Yokota, Tatsuya Okano, Shingen Misaka, Shizu Hidema, Kazuaki Kanai, Kenju Shimomura, Yuko Maejima

**Affiliations:** 1 Department of Bioregulation and Pharmacological Medicine, Fukushima Medical University School of Medicine, Fukushima 960-1295, Japan; 2 Department of Neurology, Matsumura General Hospital, Fukushima 970-8516, Japan; 3 Department of Neurology, Fukushima Medical University School of Medicine, Fukushima 960-1295, Japan

**Keywords:** glucagon, insulin, intra-islet GLP-1, oxytocin

## Abstract

Objective: Oxytocin (Oxt) is secreted to the peripheral body through the pituitary gland and can induce insulin secretion under high glucose conditions. Insulin secretion is regulated by various factors including glucagon-like peptide (GLP)-1, secreted from intestinal L-cells. GLP-1 is also expressed and secreted within islets and termed as “intra-islet GLP-1”. The study aims to elucidate the impact of Oxt on insulin secretion in relation to intra-islet GLP-1.

Methods: We measured changes in blood glucose and insulin levels following Oxt administration in wild-type (WT) and Oxt receptor knockout (OxtR KO) mice. Additionally, we assessed insulin secretion from islets isolated from WT and OxtR KO mice under conditions with and without Oxt. Histological analysis of OxtR expression in islets was performed. The effects of Oxt on factors influencing insulin secretion, such as glucagon, GLP-1 secretion from WT islets and K_ATP_ channel activity were also investigated.

Results: Oxt injection induced a temporal rise in blood glucose levels in both WT and OxtR-KO mice at 15-min post-injection. In WT mice, blood glucose level returned to control levels by 30min and were significantly lower at 60-min. OxtR KO mice maintained elevated glucose levels at 30-min. WT mice showed a significant increase in insulin levels at 15-min, while OxtR KO mice did not. OxtR was expressed in both insulin and glucagon-positive cells with higher expression in glucagon-positive cells. WT islets showed an increase in intra-islet GLP-1 secretion upon Oxt application.

Conclusions: The study indicates that Oxt may enhance insulin secretion by promoting the secretion of intra-islet GLP-1.

## INTRODUCTION

Oxytocin (Oxt) is a nine-amino-acid neuropeptide produced in the hypothalamus and secreted both within the brain and into peripheral blood circulation [1]. Therefore, Oxt exerts both central and peripheral effects, and in the past, we have focused on Oxt’s effect on metabolic regulation in both central and peripheral tissues [2–4]. The central effects of Oxt on metabolic regulation include food intake control while the peripheral effects include reduction of body fat [1]. However, the effect of Oxt on controlling blood glucose and its underlying mechanism, especially the direct effect on pancreatic islet, remains to be elucidated.

In the past, numerous studies have been conducted to clarify the effect of Oxt on insulin secretion [5–7]. The results of these studies have been contradictory, showing increases in blood glucose, insulin levels and glucagon levels [5–8]. The contradicting results may be explained by complexity of Oxt on receptor bindings. Oxt exerts its effect by binding to its receptor (OxtR), but due to its similarity to the structure of vasopressin, at high concentration Oxt can bind to vasopressin receptor [9]. We have also shown in the past that when Oxt is applied directly to isolated islets, it increases insulin section in high-glucose conditions [2]. However, insulin secretion from islet is regulated by various factors such as K_ATP_ channel, glucose metabolism, ATP production capacity, fatty acids, glucagon, and incretins such as glucagon-like peptide-1 (GLP-1) [10–13].

GLP-1 was originally discovered as a gut hormone secreted from intestinal L-cells in response to nutrients. GLP-1 is a potent incretin hormone that contributes to glucose homeostasis by promoting glucose-induced insulin secretion from pancreatic β-cells while simultaneously inhibiting glucagon secretion from α-cells [14, 15]. Once secreted from intestinal L-cells, GLP-1 is considered to reach pancreatic islets through portal circulation to promote insulin secretion [16]. The effect of incretins on potentiating insulin secretion is clinically used for the treatment of type 2 diabetes, such as GLP-1 analogues and DPP4 inhibitors which inhibit GLP-1 inactivating enzyme, DPP4 [17]. However, recent evidence suggests that α-cells in pancreatic islet also synthesize and secrete GLP-1. The α-cells derived “intra-islet GLP-1” may contribute to the insulin secretion from β-cells in a paracrine manner [18, 19]. GLP-1 is known to be cleaved from proglucagon. The proglucagon gene is expressed in both intestinal L-cells and pancreatic α-cells. The α-cells express prohormone convertase 2 (PC2) which processes proglucagon to yield glucagon while L-cells express PC 1/3 which processes and yields GLP-1 from proglucagon [20, 21]. However, human α-cells were found to be capable of producing GLP-1 and HPLC analysis of extracts from α-cells identified a small quantity but the existence of GLP-1 [22]. Also, GLP-1 was identified co-packed with glucagon granules from α-cells. These reports indicate that GLP-1 can be produced and secreted within the islet [23–25]. However, the physiological contribution of intra-islet GLP-1 to insulin secretion and the underlying mechanism that regulates intra-islet GLP-1 secretion remain unclear. Here, we show the possible involvement of Oxt in intra-islet GLP-1 secretion, which may influence the insulin secretion from islets.

## RESULTS

### Blood glucose after intraperitoneal (IP) injection of Oxt

**Figure 1:**
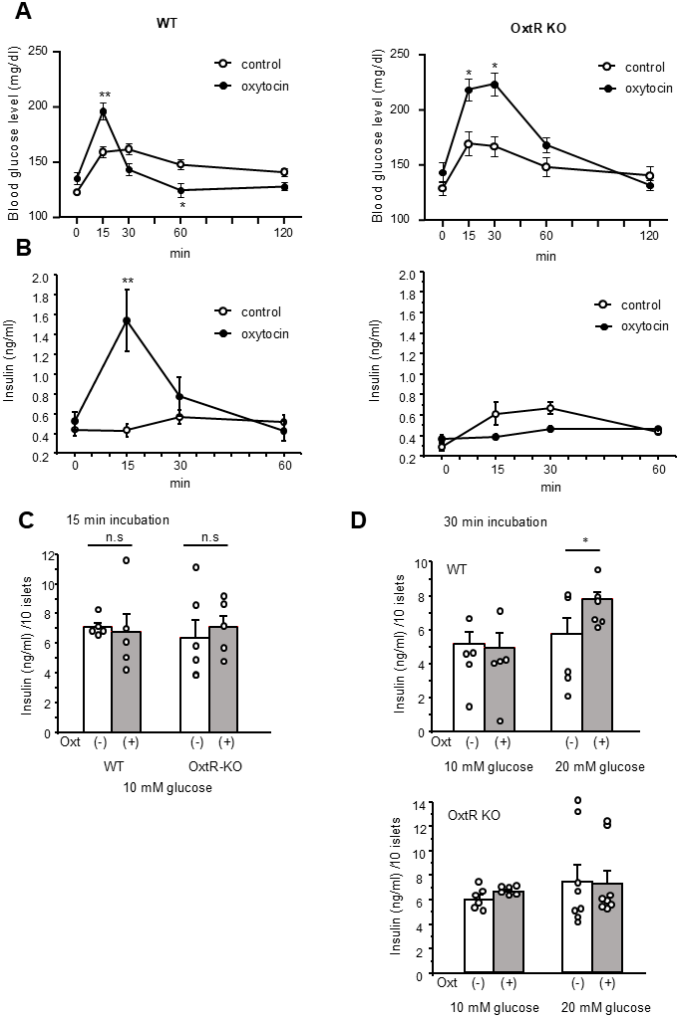
**Blood glucose change and insulin secretion after Oxt injection.** (**A**) Change of blood glucose after Oxt (400 μg/kg) injection in wild type mice (left, n = 14, 15) and OxtR KO mice (right, n = 6, 6). (**B**) Changes of insulin levels after Oxt (400 μg/kg) injection in wild type mice (left, n = 5, 5) and OxtR KO mice (right, n = 5, 5). *: p < 0.05, **: p < 0.01, repeated measures two-way ANOVA followed by Sidak’s multiple range test. (**C**) Insulin secretion from isolated islets after 15 min from wild type and OxtR KO mice (open bar: control, grey bar: with Oxt, n = 5 wells for each condition). n.s: not significant. unpaired t-test. (**D**) Top panel: Insulin secretion from isolated WT mice islets after 30 min in control medium (open bar; 10 mM glucose: n = 5 wells, 20 mM glucose: n = 5 wells) and medium with Oxt (grey bar 10 mM glucose: n = 5 wells, 20 mM glucose: n = 6 wells). Bottom panel: Insulin secretion from isolated OxtR KO mice islets after 30 min in control medium (open bar; 10 mM glucose: n = 6 wells, 20 mM glucose: n = 8 wells) and medium with Oxt (grey bar10 mM glucose: n = 6 wells, 20 mM glucose: n = 8 wells). *: p < 0.05, unpaired t-test.

In wild type mice, the IP injection of Oxt resulted in a significantly elevated blood glucose level at t = 15 min post-injection, followed by lower blood glucose level at t = 60 min compared to IP saline-injected control mice (time × treatment, F_4, 108_ = 23.86, P < 0.01) (Figure 1A). Conversely, in OxtR KO mice, the same IP Oxt injection led to a significantly higher blood glucose level at both t = 15 and 30 min compared to OxtR KO mice injected with saline (time × treatment, F_4, 40_ = 8.997, P < 0.01) (Figure 1A).

Furthermore, in wild type mice, IP injection of Oxt significantly increased insulin levels at t = 15 min compared to IP saline injected control mice (time × treatment, F_3, 27_ = 13.39, P < 0.01) (Figure 1B). However, in OxtR KO mice, Oxt treated mice showed no significant difference in insulin levels at any time point compared to OxtR KO mice injected with saline (time × treatment, F_3, 27_ = 4.39) (Figure 1B).

The results indicate that the effect of Oxt injection on increasing blood glucose levels at t = 15 min is independent of the OxtR mediated Oxt signalling pathway. Additionally, since insulin levels were increased only at 15 min after IP injection of Oxt in wild type mice, the reduction in blood glucose levels at t = 30 and 60 min observed only in wild type mice should be attributed to the elevated insulin levels induced by the Oxt IP injection.

### Effect of Oxt on insulin secretion from isolated islets

To further investigate the effect of Oxt on increasing blood glucose levels at t = 15 min in both WT and OxtR KO mice, we performed a 15-minute batch incubation under 10 mM glucose conditions with islets isolated from WT and OxtR KO mice and measured insulin secretion. As shown in Figure 1C, the insulin secretion was not affected by the presence of Oxt in both islets from WT and OxtR KO mice, indicating that the blood glucose increasing effects observed by Oxt treatment at the 15 min time point in both WT and OxtR KO mice are independent of insulin secretion.

Next, we investigated the effect of Oxt on insulin secretion after 30 min of application. After 30 min of batch incubation of WT islets under 10 mM glucose conditions, Oxt failed to increase insulin secretion compared to the control group. However, when WT islets were incubated in 20 mM glucose conditions for 30 min, Oxt significantly increased insulin secretion compared to the control (Figure 1D). In contrast, in islets isolated from OxtR KO mice, Oxt failed to increase insulin secretion in both 10 mM and 20 mM glucose condition (Figure 1D). These results indicate that Oxt enhances insulin secretion from islets only under high glucose conditions in islets from WT mice, corresponding to the blood glucose-lowering and insulin-increasing effects of Oxt observed in WT mice, but not in OxtR KO mice, as shown in Figure 1A, 1B.

### Effect of Oxt on glucagon secretion

To investigate the possible underlying mechanism of Oxt on increasing insulin secretion, we checked whether Oxt can influence the factors that may influence the insulin secretion.

**Figure 2:**
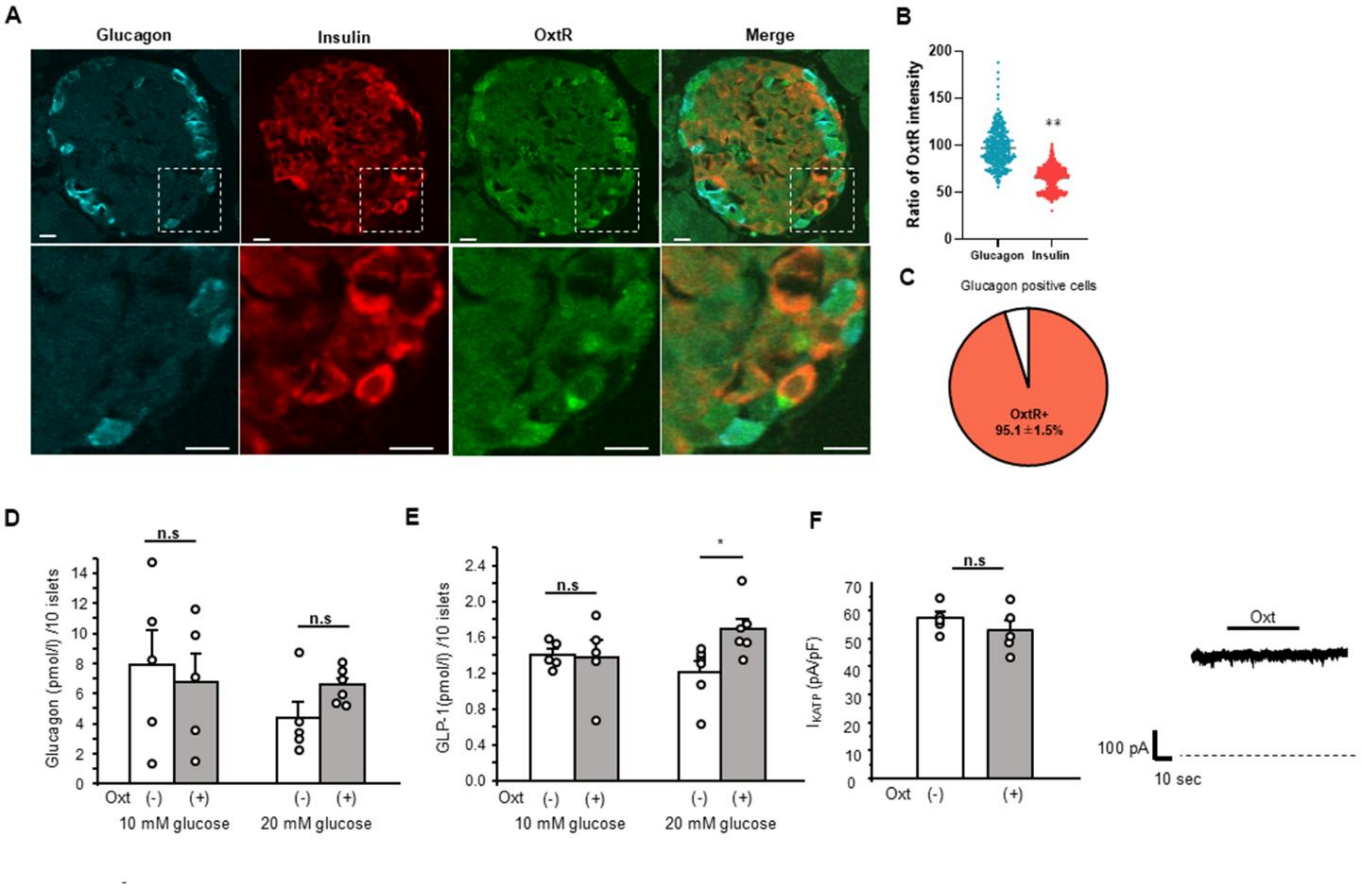
**Relation of Oxt and glucagon.** (**A**) Representative images of immunostaining for glucagon, insulin and OxtR in an islet (upper panels). Bottom panels are enlarged images of white square in each upper panel. Scale bars in the image indicate 10 μm. (**B**) The ratio of OxtR intensity in glucagon positive cells and insulin positive cells. The average intensities of OxtR in glucagon was adjusted to 100%. (Glucagon; n = 335, Insulin; n = 626). **: p < 0.01. unpaired t-test. (**C**) The pie chart shows percentage of OxtR positive cells in glucagon positive cells (n = 13 islets). (**D**) Glucagon secretion from WT mice islets after 30 min in control (open bar, 10 mM glucose: n = 5 wells, 20 mM glucose: n = 5 wells) and in the presence of Oxt (grey bar, 10 mM glucose: n = 5 wells, 20 mM glucose: n = 6 wells). n.s: not significant. unpaired t–test. (**E**) Secretion of GLP-1 from isolated WT mice islets after 30 min in control medium (open bar, n = 6 wells) and medium with Oxt (grey bar, n = 6 wells). *:p < 0.05. unpaired t-test. (**F**) Left: K_ATP_ channel current from MIN6 cells in control medium (open bar, n = 5 cells) and after application of Oxt (10^-7^ M) (grey bar, n = 5 cells). n.s: not significant. unpaired t–test. Right: The representative K_ATP_ channel current with Oxt (10^-7^ M) application. Dotted line indicates zero current level. The line on top of current indicates the Oxt application period.

First, we have investigated the possible involvement of glucagon secretion. As shown in Figure 2A, OxtR KO are expressed in both insulin and glucagon positive cells, and histological study showed the intensities of OxtR KO were higher in glucagon positive cells than insulin positive cells (Figure 2B), indicating the abundant expression of OxtR KO on glucagon secreting pancreatic α-cells. 95.1 ± 1.5 % of glucagon positive cells were found to be positive with OxtR KO (Figure 2C). Therefore, we have applied Oxt for 30 min to islets under 10 mM and 20 mM glucose conditions in islets isolated from WT mice. However, Oxt failed to affect glucagon secretion in both glucose conditions indicating that Oxt has no direct effect on glucagon secretion (Figure 2D), regardless of glucose level.

### Effect of Oxt on GLP-1 secretion

Next, we investigated the effect of Oxt on GLP-1 secretion from islets isolated from WT mice. When performing the GLP-1 secretion analysis with batch incubation of isolated islets, Oxt showed no effect under 10 mM glucose condition but significantly increased GLP-1 secretion after 30 min of incubation under 20 mM glucose conditions (Figure 2E).

### Effect of Oxt on K_ATP_ channel activity

Another factor that affects the insulin secretion within short period such as 15-30 min is the ATP sensitive potassium (K_ATP_) channel. Therefore, we measured the activity of the K_ATP_ channel using patch-clamp technique and investigated whether Oxt has direct effect on K_ATP_ channel activity.

As shown in Figure 2F, we checked in five different MIN6 cells, but Oxt failed to show any effect on K_ATP_ channel activity (1.16 ± 1.2x compared to control current level).

## DISCUSSION

Oxt is reported to have various effects on controlling body metabolism, including insulin secretion and blood glucose regulation [2, 5–8].

Interestingly, we found that blood glucose levels increase after Oxt IP injection in both WT and OxtR KO mice. Several previous studies reported that IP injection of Oxt increases blood glucose levels [5, 6]. However, by using OxtR KO mice, our present study demonstrated that insulin levels are increased after IP injection of Oxt in WT mice but not in OxtR KO mice and increased blood glucose after IP injection of Oxt is likely to be independent of the OxtR system. Further study is required to clarify the underlying mechanisms.

In our previous studies, we demonstrated that Oxt can enhance insulin secretion from islets exclusively under high glucose conditions within 30 min period [2]. The mice we utilized in this study were C57BL/6J mice, including OxtR KO mice in the same genetic background. Given that C57BL/6J mice are known to exhibit higher postprandial and free-fed blood glucose level compared to other strains such as DBA/2, BALB/C and C3H/HeH [26], we investigated the effect of Oxt on insulin secretion from isolated islets at 20 mM glucose as high glucose condition. Consistent with our previous findings, Oxt increased insulin secretion from islets under 20 mM glucose conditions. However, islets isolated from OxtR KO mice did not exhibit this effect, indicating that Oxt indeed enhances insulin secretion from islets in high glucose condition. The precise mechanism underlying Oxt-induced insulin secretion remains incompletely understood. It is generally accepted that Oxt stimulates its G-protein coupled receptor expressed in pancreatic β-cell, leading to an increase in intracellular calcium levels and enhancing glucose-stimulated insulin secretion [27]. Therefore, the physiological function of Oxt may be, to amplify the insulin secretion pathway, similar to that of GLP-1. However, as demonstrated in this study, OxtR is mainly expressed in glucagon-producing α-cells with significantly lower expression in insulin-secreting β-cells. Consequently, it can be inferred that the enhancement of insulin secretion by Oxt is not solely due to its direct effect on β-cells but also through an indirect effect on α-cells.

The main contribution of α-cells to blood glucose is by secreting glucagon. However, in this study, we have confirmed that glucagon secretion from islet was not affected by Oxt and although the difference was not statistically significant, glucagon secretion tended to be enhanced by Oxt in high glucose conditions, which is contradicting to the previous report showing that Oxt increases insulin secretion and reducing blood glucose levels [2].

By using antibodies and ELISA assays that are highly specific to GLP-1, we have found that intra-islet GLP-1 secretion is increased from islets in the presence of Oxt.

The presence of intra-islet GLP-1 was once controversial, but it is now widely accepted that rodent and human α-cells can produce and secrete GLP-1 [18]. This conclusion is supported by numerous reports showing evidence of existing GLP-1 in both human and rodent islets [18, 22–25].

GLP-1 in pancreatic islets is produced primarily in α-cells by tissue-specific post-translational processing of proglucagon, a peptide hormone derived from the proglucagon gene [28]. This cell-specific processing in GLP-1 production is primarily due to the action of enzymes PC1/3, while another enzyme, PC2, cleaves proglucagon to glucagon. In α-cells, PC2 dominates proglucagon processing, with glucagon being the major secretory product [20, 21]. However, under certain conditions, α-cells can express PC1/3 and can shift proglucagon processing towards GLP-1 production [20, 21].

The functional contribution of intra-islet GLP-1 remains unclear. Intra-islet GLP-1 is considered to stimulate insulin secretion similar to L-cell derived GLP-1 [18, 29, 30]. There is substantial evidence suggesting that glucose acts as a pivotal regulator of intra-islet GLP-1 production and secretion. *In vitro* studies have consistently demonstrated that in primary islets in culture, they exhibit greater GLP-1 secretion when exposed to higher glucose concentrations in the medium [31, 32]. Our current findings also corroborate these observations revealing that GLP-1 secretion from WT islets is markedly enhanced under high glucose conditions (20 mM glucose). These results, along with previous reports, indicate that intra-islet GLP-1 plays a crucial role in stimulating insulin secretion in high glucose conditions.

These results indicate the possibility of a new concept for insulin secretion stimulation in which Oxt stimulates intra-islet GLP-1 secretion from α-cells and this secreted intra-islet GLP-1 stimulates insulin secretion from β-cells in paracrine manner. Although the detailed mechanism for the stimulation of intra-islet GLP-1 secretion remains to be elucidated and further studies are required, as far as we know, our present study is the first to report the effect of Oxt on inducing intra-islet GLP-1 secretion.

Normalization of insulin secretion is important for elder type 2 diabetes patients. Due to the recent focus on GLP-1 and its analogues for treatment, our present study suggests a new possibility for Oxt to induce internal intra-islet GLP-1 secretion, which may enhance insulin secretion and thus hold potential for diabetes treatment.

## MATERIALS AND METHODS

### Animals

Male C57BL/6J mice (aged 10-15 weeks); Japan SLC Shizuoka Japan, male OxtR-deficient mice (OxtR KO) [32] (aged 10-15 weeks) and heterogeneous OxtR-Venus knock in (OxtR Venus/+) mice [33, 34] were used in this study. The mice were maintained on a 12-hour light/dark cycle (light on from 07.00-19.00). Mice were allowed ad libitum access to water and a standard diet (CE-2; Clea, Osaka, Japan). All experimental procedures and care of animals were carried out according to the relevant guidelines and regulations, and were approved by the Fukushima Medical University Institute of Animal Care and Use Committee.

### Blood glucose and insulin measurements

Mice were habituated for 7 days before experiment. Following a 4-hour food deprivation after onset of light phase, the animals received intraperitoneally (IP) injection of saline (control) or 400 μg/kg Oxt (Peptide Institute, Osaka, Japan). The dose of Oxt was selected based on our previous research, which demonstrated significant metabolic difference following IP injection [2]. Blood was sampled by cutting tip of tail without restraint, and blood glucose levels were measured using glutest aqua (AEKRAY, Kyoto, Japan) at 0, 15, 30, 60 and 120 min after IP injection.

For insulin measurement, 30 μl blood samples were collected by pipette from cutting tip of tail at 0, 15, 30 and 60 min after IP injection of Oxt (400 μg/kg). In this sampling, EDTA coated pipette tips were used. Blood samples were immediately centrifuged at 3000 rpm for 10 min at 4° C. Supernatant was collected and stored -80° C, until measurement. Insulin, was measured using Morinaga Insulin ELISA assay kit (Morinaga, Yokohama, Japan).

### Insulin, glucagon, GLP-1 secretion

After isolation, islets were cultured overnight in DMEM medium before insulin secretion was assessed. After 1 h of starvation in 2 mM glucose, insulin secretion was measured during static incubations (10 islets/well) in 2 ml Krebs-Ringer Buffer (KRB). The KRB solution contained (in mM) 118.5 NaCl, 2.54 CaCl_2_, 1.19 KH_2_PO_4_, 4.74 KCl, 25 NaHCO_3_, 1.19 MgSO_4_, and 10 HEPES (pH 7.4 with NaOH) with 0.1% bovine serum albumin. Islets were incubated with 10^-7^ M for 15 or 30 minutes. Samples of the supernatant were assayed for insulin. Insulin, was measured using Morinaga Insulin ELISA assay kit (Morinaga, Yokohama, Japan). Glucagon and GLP-1 were measured using Fujifilm ELISA assay kit (Fujifilm Wako, Tokyo, Japan).

### Triple immuno-staining glucagon, insulin and OxtR

The OxtR Venus/+ mice were perfused with 4% paraformaldehyde and 0.2% picric acid, and pancreases were removed. Paraffin-embedded pancreas sections from OxtR Venus/+ (n = 3) mice were deparaffinized with xylene. The slide sections were then washed in PBS (0.01 M. pH 7.4) and incubated with blocking solution (0.1% triton-X, 2% bovine serum albumin (BSA) and 5% normal goat serum (NGS)) for 1 hr. Next, the sections were incubated with monoclonal mouse anti-glucagon antibody (1:2000, G2654, Sigma-Aldrich, MO, USA), guinea pig anti-insulin antibody (1:3, A0564, Dako-Agilent, CA, USA) and rabbit anti-GFP antibody (1:1000, A11122, Thermo Fisher Scientific, IL, USA) for overnight at 4° C. The sections were rinsed in PBS and incubated with Alexa 405 labelled goat anti-mouse IgG (1:500, Thermo Fisher Scientific, IL, USA), Alexa 594 labelled goat anti-guinea pig IgG (1:500, Thermo Fisher Scientific, IL, USA), and Alexa488 labelled goat anti-rabbit IgG in PBS containing 2% BSA and 5% NGS for 40 min. Next, the sections were rinsed with PBS and covered with mounting medium containing DAPI (4′,6-diamidino-2-phenylindole) (Vector Laboratories, CA, USA). Fluorescence images of islets were acquired with a confocal laser-scanning microscope (FV10i; Olympus, Tokyo, Japan). The intensity of OxtR fluorescence in glucagon and insulin positive cells were analyzed, respectively by NIH image software (Image J, National Institute of Health). 6 islets from each mouse (n = 3) were analyzed.

For analysis of colocalization of glucagon positive cells and OxtR positive cells, glucagon positive cells and OxtR positive cells were counted from confocal images. The percentages of OxtR positive cells in α cell were calculated. Thirteen islets from three mice were analyzed.

### Electrophysiological analysis

The K_ATP_ channel currents were recorded at room temperature (22–25° C) using the whole cell patch clamp method with an Axopatch 200B amplifier (Axon Instruments Inc., CA, USA) controlled by Clampex 10.2 software via a Digidata 1320A interface (Molecular Devices, CA, USA). The standard extracellular solution contained 5.6 mmol/L KCl, 138 mmol/L NaCl, 2.6 mmol/L CaCl_2_, 1.2 mmol/L MgCl_2_, and 10 mmol/L HEPES. The pH value of extracellular solution was adjusted to 7.4 with NaOH. The pipette solution contained 107 mmol/L KCl, 1 mmol/L CaCl_2_, 1 mmol/L MgCl_2_, 10 mmol/L HEPES and 10 mmol/L EGTA. 0.3 mmol/L ATP was added to prevent rundown^17^. The pH value of the pipette solution was adjusted to 7.2 with KOH. Glass pipettes were prepared from borosilicate tube glass (Narishige, Tokyo, Japan) and had 2-5 MΩ when filled with the pipette solution. Following gigaohm seal formation, negative pressure was applied to the pipette to rupture the membrane and establish the whole-cell mode. The K_ATP_ currents were measured in response to holding potential of -60 mV. The recorded currents were confirmed to be K_ATP_ channel current by application of sulfonylurea tolbutamide (500 μM) at the end of experiments. The application of an extracellular solution containing 20 mM glucose had no effect on K_ATP_ channel current, indicating that the intracellular complex, including the glycolysis system, is replaced by the pipette solution. Therefore, factors such as glucose metabolism do not interfere with the K_ATP_ channel under our experimental conditions. Data acquisition and storage were conducted with the use of a pClamp 10.2 (Molecular Devices, CA, USA).

### Statistical analysis

All data are expressed as mean ± SEM. The comparison of data from two groups was performed using Student’s t-test. The change of blood glucose after IP injection of OXT were analyzed by repeated measures two-way ANOVA followed by Sidak’s multiple range test. All statistical tests were two-tailed, with values of p < 0.05 considered statistically significant.
